# Eco-Evolutionary Thinking in Metastatic Breast Cancer: A Clinician’s Primer on Why Tumors Outsmart Us and How to Fight Back Smarter

**DOI:** 10.3390/cancers18182979

**Published:** 2026-09-15

**Authors:** Aixa Elena Soyano, Renee Brady-Nicholls, Hatem H. Soliman, Robert A. Gatenby, Mark Robertson-Tessi, Dana Ataya

**Affiliations:** 1 Department of Breast Medical Oncology, Moffitt Cancer Center, Tampa, FL 33612, USA; 2 Integrated Mathematical Oncology (IMO) Department, Moffitt Cancer Center, Tampa, FL 33612L, USA; 3 Diagnostic Imaging and Interventional Radiology Department, Moffitt Cancer Center, Tampa, FL 33612, USAdana.ataya@moffitt.org (D.A.)

**Keywords:** breast cancer, metastatic breast cancer, eco-evolutionary, adaptive therapy

## Abstract

Metastatic breast cancer (MBC) remains incurable because of tumor evolution. Despite advances in targeted therapies—CDK4/6 inhibitors, PIK3 inhibitors, HER2-directed agents, and antibody–drug conjugates—median overall survival is modest, with resistance emerging in nearly all patients. From an evolutionary perspective, standard continuous maximum tolerated dose (MTD) therapy may create intense selective pressure that reduces treatment-sensitive cells and, in some incurable and resistance-prone settings, could permit competitive release of resistant clones. While MTD remains the guideline-endorsed standard of care in many metastatic settings and has produced substantial survival gains, the eco-evolutionary framework highlights a potential trade-off rather than a categorical failure of MTD. Cancer is not just a genetic disease but an evolving ecosystem where cell populations compete for limited resources. Resistant cells typically carry a fitness cost—slower growth in the absence of treatment—creating an exploitable vulnerability. By preserving treatment-sensitive cells through dose modulation or treatment holidays (adaptive therapy), we can harness competitive suppression to control resistant populations and prolong disease control, potentially doubling time to progression compared to continuous MTD. This primer translates these ideas into plain language for clinical trial application. We explain four core eco-evolutionary principles every breast oncologist should know, review emerging evidence from adaptive therapy trials in breast and other cancers, and discuss practical implementation strategies to incorporate into future trials. The goal is not cure through eradication, but long-term control through evolutionary management. These evolution-informed strategies may, in selected contexts, help delay resistance and prolong disease control, and represent a complementary framework that warrants prospective testing.

## Simple Summary

Breast cancer that has spread to other parts of the body usually cannot be cured, because the cancer cells change over time and become resistant to treatment. Doctors currently try to kill as many cancer cells as possible using the highest safe drug doses, but this approach can backfire, wiping out the drug-sensitive cells that would otherwise hold the resistant cells in check, allowing resistance to grow faster. This article introduces a different way of thinking about cancer, borrowed from ecology, where cancer is viewed as a population of competing cells rather than simply a genetic problem. By adjusting drug doses or timing to preserve some treatment-sensitive cells, doctors may be able to keep resistant cells suppressed for longer. We explain the key ideas behind this approach in plain language, review early evidence from clinical trials, and discuss how blood tests can help track these changes over time.

## 1. Introduction

Most patients with metastatic breast cancer (MBC) respond favorably… at first. When we start a patient on first-line therapy for newly diagnosed hormone receptor positive (HR+)/HER2 negative MBC, we typically see tumor shrinkage, symptom improvement, and normalization of tumor markers. The median progression-free survival (PFS) for a combination of CDK 4/6 inhibitors with endocrine therapy in the first line is around 24 months [1,2,3]. For HER2+ disease treated with pertuzumab, trastuzumab, and chemotherapy, it is around 18 months [4]. These are real gains that have meaningfully improved patient lives. Unfortunately, resistance is inevitable, even with “targeted” or “precision” drugs. Despite initial responses, progression is nearly universal. In a modeled US cohort analysis, median overall survival (OS) for all MBC patients was only 3.2 years, ranging from just 1.6 years for triple-negative disease to 4.9 years for HR+/HER2+ disease, though survival varies considerably by geography, treatment era, and subtype and is improving in several subgroups [5]. Even the 20–30% of patients who survive five years or longer, most eventually progress [6]. The problem is not that our drugs do not work. They clearly do work, at least initially. The problem is that tumors evolve around them.

CDK4/6 inhibitors double PFS but almost never cure. The addition of CDK4/6 inhibitors to endocrine therapy represents one of the most significant advances in metastatic HR+ HER2 negative breast cancer treatment in the past decades. Yet despite this dramatic benefit, CDK4/6 inhibitors are not curative. Resistance develops through multiple mechanisms, including RB1 loss, activation of alternative growth pathways, and cell cycle alterations [7,8].

The question we all ask: “Why can’t we stop the cancer from coming back?” The answer lies in understanding cancer not just as a genetic disease, but as an evolutionary and ecological process. Every tumor contains heterogeneous populations of cells with different genetic and phenotypic characteristics [9,10]. When we treat with a continuous maximum tolerated dose (MTD), we may create intense selective pressure that favors the survival and proliferation of resistant variants [11,12]. By eliminating the sensitive population, we remove the competitive suppression that was keeping resistant cells in check, a phenomenon called competitive release [13,14]. The resistant cells, now freed from competition, expand rapidly to cause clinical progression.

This evolutionary perspective fundamentally reframes how we should think about treatment. If resistance is inevitable due to selection on pre-existing or rapidly arising variants, then our goal cannot be complete eradication in most cases. Instead, we need strategies that manage tumor evolution: maintaining sensitive populations to suppress resistant ones, exploiting fitness costs of resistance, and using treatment strategically rather than continuously [12,15,16]. This is the promise of eco-evolutionary thinking in MBC.

The aim of this primer is to offer a conceptual framework for integrating eco-evolutionary thinking into breast oncology, not to define new standards of care. It is distinguished from prior eco-evolutionary reviews by its clinician-facing, breast-specific focus and its integration with current breast cancer trials.

## 2. Treatment of Breast Cancer over Time

Over the past three decades, the treatment of MBC has evolved through successive therapeutic paradigms that reflect increasing biological specificity but reveal a consistent pattern of transient disease control. HR+ HER2 negative MBC has progressed from single-agent endocrine therapy with tamoxifen or aromatase inhibitors, to combination strategies incorporating CDK4/6 inhibitors, and more recently to selective estrogen receptor degraders [1,2,3,15,17]. Each step has improved PFS and, in some settings, OS, yet none has eliminated the near inevitability of acquired resistance.

A similar evolution is observed in HER2+ disease, beginning with trastuzumab monotherapy, advancing to dual HER2 blockade with pertuzumab, followed by antibody–drug conjugates (ADCs) such as T-DM1 and, more recently, trastuzumab deruxtecan [18,19,20]. Each innovation has produced deeper and more durable responses than its predecessor, but sequential relapse remains the dominant clinical outcome.

In triple-negative breast cancer (TNBC), where stable oncogenic dependencies are less common, chemotherapy has remained foundational, with immune checkpoint inhibitors and ADCs providing incremental benefit in selected populations rather than durable disease eradication [21,22].

Across subtypes, the unifying lesson from decades of landmark trials is not therapeutic failure but biological inevitability: strong initial responses followed by adaptive escape. These repeated clinical patterns—tumor regression under therapy, followed by progression through resistant disease—highlight MBC as an evolving system shaped by selective pressures, clonal competition, and ecological constraints [23]. This perspective motivates the need to move beyond static treatment strategies toward frameworks that explicitly account for tumor evolution over time.

## 3. Cancer as an Ecosystem in Darwinian Evolution

Every oncologist has seen it: a patient’s tumor shrinks beautifully on scans, tumor markers drop, symptoms improve—and then, months later, the cancer roars back, now unresponsive to the same drug that worked so well initially. One explanation focuses on genomic mutations and resistance to drugs, but the eco-evolutionary lens complements this understanding by considering the ecological interactions among tumor subpopulations. Tumors are not monolithic masses of identical cells; they are diverse ecosystems containing multiple competing subpopulations, or subclones, each with different sensitivities to treatment [24,25].

Even before treatment starts, resistant cells are often already present in small numbers [26]. These pre-existing resistant populations arise through the tumor’s inherent genetic and epigenetic instability; up to 63% of somatic mutations can be heterogeneous within individual tumors [10]. This intratumoral heterogeneity means that when we biopsy a tumor, we are sampling just one neighborhood of a complex, spatially structured community where different clones occupy different niches [25,27].

Think of a tumor like a forest with different tree species. Some trees (sensitive cells) grow rapidly and dominate the canopy when conditions are favorable. Others (resistant cells) grow more slowly but can tolerate harsh conditions—drought, fire, or, in our case, chemotherapy. Before treatment, the fast-growing sensitive cells vastly outnumber the resistant ones, simply because they are better adapted to the untreated tumor environment [12].

## 4. Maximum Tolerated Dose Chemotherapy

MTD chemotherapy, the standard approach we have used for decades, operates on a simple principle: kill as many cancer cells as possible, as quickly as possible. This strategy makes intuitive sense and often produces dramatic initial responses. While MTD remains the guideline-endorsed standard of care in many metastatic settings and has produced substantial survival gains, from an evolutionary perspective, MTD therapy may be suboptimal in specific settings where resistant cells are already present and cure is not achievable [11].

When we administer MTD therapy, we eliminate most treatment-sensitive cells. These were the cells that were outcompeting the resistant cells for space, nutrients, and growth factors. By removing them, we inadvertently eliminate the very population that was keeping resistant cells in check. This phenomenon of competitive release is well-documented in nature [11,14,28]. When wolves were eliminated from Yellowstone, elk populations exploded because their primary predator was gone. When we eliminate sensitive cancer cells with aggressive chemotherapy, resistant cells experience the same explosive growth—not because they suddenly became more aggressive, but because we removed their competition [11,13,14]. The resistant cells, previously suppressed by faster-growing sensitive cells, now have access to unlimited resources and space. They proliferate rapidly, and the tumor relapses in a resistant form [11,29].

While the competitive-release framework offers a biologically plausible explanation for treatment failure under continuous MTD, it is important to emphasize that this remains a hypothesis-generating concept rather than an established clinical principle in MBC. Much of the supporting evidence derives from natural observations, mathematical models, preclinical systems, and studies in other malignancies, and the extent to which competitive release governs resistance in any individual patient’s tumor is not yet defined.

## 5. Fitness Cost of Sensitive and Resistant Cells

A central premise from evolutionary biology is that resistance often, but not universally, carries a fitness cost [11,30,31]. Resistant cancer cells must divert energy and resources to maintain their resistance mechanisms, whether that means overexpressing drug efflux pumps, activating alternative signaling pathways, or maintaining DNA repair machinery. These adaptations consume cellular resources that would otherwise be available for proliferation [31].

In the absence of drugs, resistant cells are typically less fit than sensitive cells [11,30,32]. Studies across multiple cancer types demonstrate this principle. In breast cancer, cells resistant to chemotherapy through upregulation of drug efflux pumps grow more slowly than sensitive cells when the drug is absent, because maintaining those pumps requires substantial metabolic energy [31]. In ovarian cancer, platinum-resistant cells exhibit diminished fitness when carboplatin is withdrawn, showing reduced proliferation and increased apoptosis compared to sensitive cells [33].

This fitness cost creates an opportunity. In the untreated tumor microenvironment (or during drug holidays), sensitive cells outcompete resistant cells because they proliferate faster and use resources more efficiently [12,32]. Spatial competition experiments demonstrate that when resistant cells must compete with sensitive cells for limited space and nutrients, the resistant population remains suppressed even though it possesses the molecular machinery to survive drug treatment [29,32].

The fitness cost is not uniform across all resistance mechanisms. Some forms of resistance, such as some estrogen receptor 1 (ESR1) and Phosphoinositide-3-Kinase (PIK3) mutations in HR+/HER2 negative MBC, may carry minimal fitness costs, while others, such as metabolic reprogramming or activation of stress response pathways, impose substantial burdens [30]. Understanding the specific fitness cost in a patient’s tumor is crucial for predicting whether alternative therapy will succeed [33,34].

This brings us to the central, counterintuitive principle of adaptive therapy: we can control cancer longer by treating less aggressively [8,12,15]. Instead of trying to kill every cancer cell with MTD, adaptive therapy deliberately maintains a population of treatment-sensitive cells. These sensitive cells act as a “biological police force,” suppressing resistant cells through competition for limited resources [11,12].

The ecological analogy in nature is predator–prey dynamics but inverted. The predators (wolves) control prey (elk) populations. In adaptive therapy, the “prey” (sensitive cells) controls the “predator” (resistant cells) by outcompeting them when drug pressure is reduced [12,15]. When tumor burden increases due to sensitive cell regrowth, we reintroduce treatment to reduce the sensitive population—but we stop before eliminating them entirely. When tumor burden decreases to a predetermined threshold, we withdraw treatment again, allowing sensitive cells to regrow and continue suppressing resistant cells [12,14,15].

This creates a stable oscillation: treatment on, sensitive cells decline and tumor shrinks; treatment off, sensitive cells regrow faster than resistant cells and suppress them through competition; repeat [12,35,36]. The tumor burden cycles within a controlled range, and the resistant population never achieves the explosive growth that leads to treatment failure [15,16].

Preclinical studies in breast cancer, ovarian cancer, and melanoma demonstrate similar principles [31,33,35]. In each case, maintaining tumor heterogeneity and exploiting competitive interactions between sensitive and resistant populations extends control far beyond what maximum-dose therapy achieves [16,29,37]. The key is recognizing that cancer is not just a genetic disease, it is an ecological and evolutionary process, and we can steer that evolution to our advantage by understanding the rules of competition in the tumor ecosystem [12,38].

## 6. Eco-Evolutionary Principles for Breast Oncologists

### 6.1. Adaptive Therapy

For decades, oncology has favored continued MTD to eliminate as many cancer cells as possible. However, when cure is not achievable—as is the case for most MBC—MTD may accelerate resistance by eliminating the treatment-sensitive cells that competitively suppress resistant populations [12].

**Figure 1:**
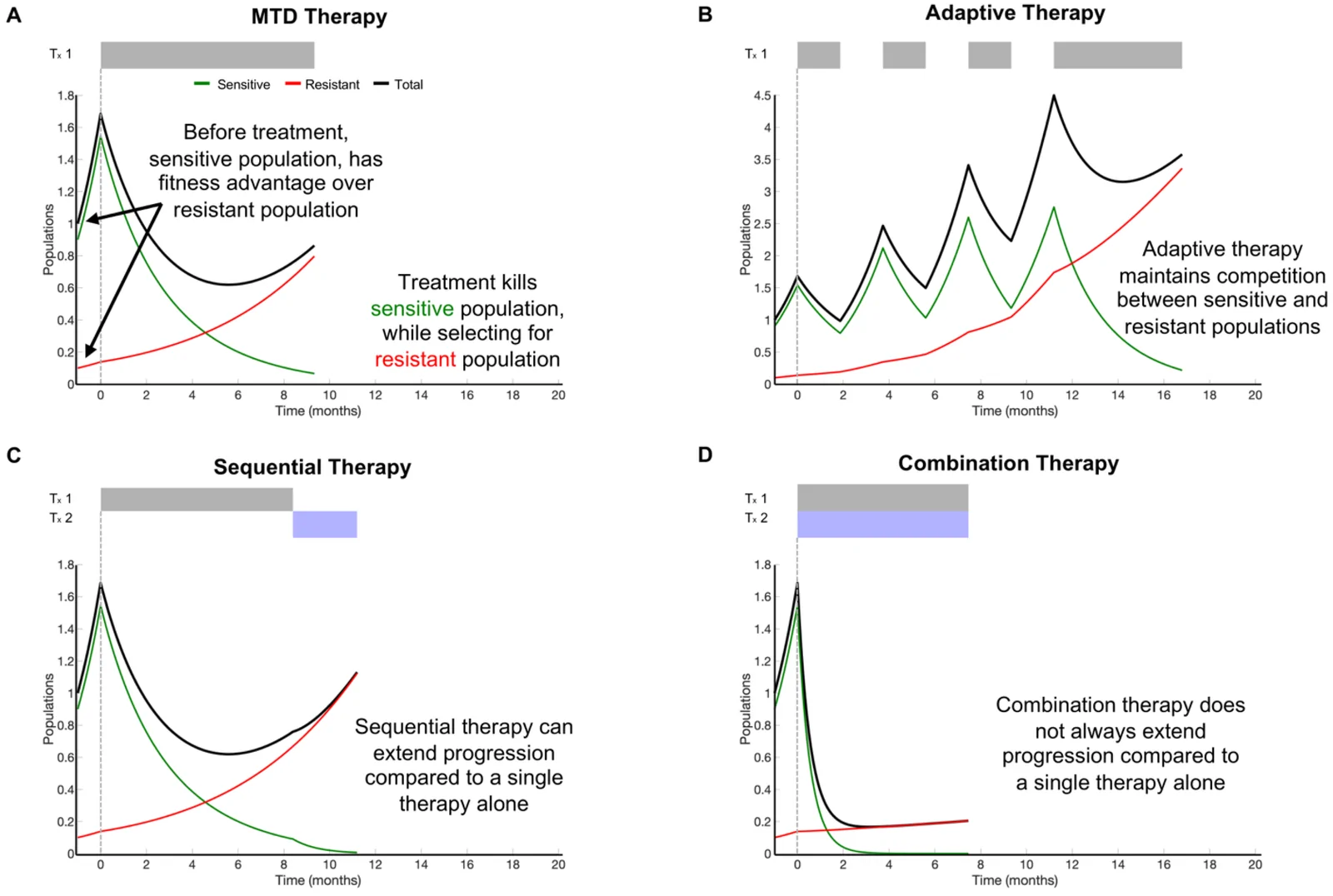
Comparison of traditional MTD and eco-evolutionary treatment strategies. (**A**) Prior to treatment, the sensitive population has a fitness advantage over the resistant population. Once treatment begins, the sensitive population declines, while the resistant population continues to grow. Eventually, the resistant population outgrows the sensitive population, resulting in treatment progression. (**B**) Treating adaptively, whereby treatment is cycled on and off to maintain the competition between the populations, can extend time to progression. (**C**) Treating sequentially, one treatment after another, can extend time to progression. (**D**) Treating concurrently with both agents may or may not extend time to progression, depending on whether the treatments are synergistic or antagonistic (shown in this case). Sensitive, resistant, and total populations are shown by the green, red, and black curves, respectively. Note that these are model simulations, meant to represent dynamics for a theoretical patient. The initial population sizes and model parameters are the same for all simulations. Model outcomes are dependent on the initial conditions and parameters chosen and are for illustrative purposes only. Relative population size is shown on the *y*-axis and time (in months) on the *x*-axis.

The key insight is competitive suppression: without treatment, sensitive cells outcompete resistant cells because resistance carries fitness costs [33,34] (Figure 1A). Resistant cells expend energy maintaining drug efflux, DNA repair, or altered metabolism—investments that reduce their proliferative capacity [33,34]. Continuous MTD eliminates sensitive cells, removing this competitive brake and allowing resistant cells to expand through competitive release [13,14] (Figure 1A).

Adaptive therapy exploits this dynamic by modulating treatment to maintain sensitive cells. When therapy is paused after tumor reduction, it allows sensitive cells to re-expand and suppress resistant cells. Treatment is resumed when tumor burden increases, preventing uncontrolled growth while preserving the competitive balance [12] (Figure 1B). Mathematical models and preclinical studies predict this approach might be able to extend time to progression compared to continuous MTD [39].

In breast cancer, preclinical studies using endocrine-resistant MCF7 xenografts showed that adaptive therapy with capecitabine and gemcitabine significantly increased survival compared to MTD, with the benefit most pronounced when drugs were alternated rather than combined [40]. Importantly, adaptive therapy resulted in tumors with fewer proliferating cells and more apoptotic cells despite lower cumulative drug doses. Clinical evidence is also emerging. In metastatic castration-resistant prostate cancer (mCRPC), adaptive abiraterone therapy in a single-institution clinical trial achieved median time to progression of 27 months compared to 14–16 months with continuous therapy in historical controls, while reducing cumulative drug exposure by 47% [41]. In breast cancer, two ongoing clinical trials are investigating adaptive therapy: one in HR+/HER2 negative MBC using capecitabine (NCT NCT06525766 (https://www.clinicaltrials.gov/study/NCT06525766 accessed on 25 May 2026)) and another in HER2 positive MBC using circulating tumor DNA (ctDNA) to guide treatment timings (NCT07459673).

The investigational implication: for patients with MBC who achieve disease control on first-line therapy, treatment holidays or dose reductions rather than continuing full-dose therapy indefinitely can be considered in clinical trials. Within such protocols, the goal shifts from “treat until progression” to “maintain stable disease,” using parameters (imaging, tumor markers, ctDNA) to guide treatment resumption [16].

#### Operationalizing Adaptive Therapy

A question from clinicians is how, and in whom, adaptive therapy would be delivered. No adaptive dosing schedule is standard of care in MBC; the parameters below are drawn from early-phase cross-tumor trials and modeling studies in breast and non-breast cancers and are presented to illustrate how such protocols are constructed, not as a treatment recommendation for routine practice.

Candidate characteristics. Adaptive protocols to date have selected patients who (i) have measurable or otherwise quantifiable disease that can be tracked longitudinally, (ii) have demonstrated an initial response to the index agent, and (iii) are not in a rapidly progressive, visceral-crisis state in which delayed dose escalation would be unsafe. In the mCRPC pilot trial of adaptive abiraterone, patients were required to have achieved a >50% decline from their pre-treatment PSA before entering the cycling strategy [42]. Translating this to MBC, plausible candidates are patients who achieve disease control on induction therapy and who have a reliable longitudinal biomarker; conversely, aggressive, high-kinetics disease and disease driven by low-cost or reversible resistance are expected to be poor candidates (see below).

Metrics to pause and resume treatment. Published protocols have used biomarker- or imaging-anchored thresholds rather than fixed calendar schedules. Molecular progression by ctDNA has been shown to precede radiographic progression by a median of roughly 3–5.8 months, which is the lead time a ctDNA-guided pause/resume rule would in principle exploit. Since MBC lacks a single universal quantitative marker, adaptive triggers would likely integrate several inputs: (i) tumor burden on imaging (RECIST-type change); (ii) conventional serum markers (e.g., CA 15-3); and/or (iii) ctDNA metrics, including mean variant allele frequency, ctDNA/tumor-fraction clearance versus persistence, and emergence of resistance alterations such as ESR1 or RB1. Exact cutoffs remain investigational and are not standardized or validated for treatment modulation.

### 6.2. Sequencing Therapy

Eco-evolutionary thinking reveals that which drugs and in what order we use them is important. The treatment sequence shapes the evolutionary trajectory of the tumor, determining which resistant clones emerge and how quickly they expand [43] (Figure 1C).

Evolutionary steering recognizes that treatment creates selective pressure that drives clonal evolution in predictable directions [44,45]. By carefully choosing the sequence and timing of therapies, we can steer evolution toward less aggressive or more treatable resistant states. Conversely, poor sequencing can inadvertently select for highly resistant, aggressive clones that are difficult to treat with subsequent therapies.

One key consideration is maintaining clonal diversity rather than driving the tumor toward a single resistant clone. Allowing some tumor heterogeneity may be beneficial because diverse populations compete with each other, preventing any single resistant clone from dominating [11].

Collateral sensitivity—the phenomenon where resistance to one drug creates hypersensitivity to another—offers opportunities for strategic sequencing [46,47]. For example, cells that develop resistance to one class of agents may upregulate metabolic pathways or stress responses that make them vulnerable to other drugs [48,49].

Temporal dynamics also matter. Studies in leukemia demonstrated that temporal collateral sensitivity (transient windows of vulnerability that appear during evolution of resistance) can be exploited by switching therapies at strategic timepoints [45]. Drug holidays may allow resistant populations to lose their resistance mechanisms if they carry fitness costs, potentially resensitizing tumors to previously failed therapies [50].

The practical implication: when sequencing therapy, consider not only efficacy but how each treatment shapes tumor evolution. Avoid sequences that promote cross-resistance. Alternate drug classes when possible, rather than exhausting one class, and use treatment holidays strategically to potentially reverse resistance.

### 6.3. Combination Therapy

A fundamental evolutionary principle is that multiple simultaneous mutations are rare. While single mutations conferring resistance to individual drugs may pre-exist in large tumor populations, cells carrying dual resistance mechanisms are less common [51]. This supports combination therapy if drugs require independent resistance mechanisms.

If two drugs share resistance pathways (e.g., the same efflux pump), one mechanism can defeat both, providing no evolutionary advantage (Figure 1D). However, drugs requiring distinct mechanisms—different targets, pathways, or adaptations—make simultaneous resistance far less likely.

This explains why some combinations succeed. Pertuzumab plus trastuzumab target different epitopes on HER2, requiring distinct resistance mechanisms [52]. Similarly, endocrine therapy plus CDK4/6 inhibitors target two different pathways (ER signaling and cell cycle regulation), reducing the likelihood of dual resistance [8].

Mathematically, double resistance is the product of individual resistance probabilities. If resistance to drug A occurs in 1 in 10^6 cells and resistance to drug B occurs in 1 in 10^6 cells, then double resistance occurs in approximately 1 in 10^12 cells—a frequency that may not exist even in large metastatic tumors [53]. This creates a therapeutic window where combination therapy can achieve durable control.

However, caveats exist. First, resistance mechanisms are not always independent; some genetic changes (e.g., TP53 mutations, chromosomal instability) increase the overall mutation rate, making multiple resistance mechanisms more likely [10]. Second, non-genetic mechanisms of resistance (phenotypic plasticity, epigenetic changes) can arise more rapidly than genetic mutations and may confer resistance to multiple drugs simultaneously [54,55]. Third, if we use combinations at MTD continuously, we still drive selection toward the rare double-resistant clones, eventually leading to treatment failure.

The investigational implication: prioritize combinations with non-overlapping resistance mechanisms. Use adaptive dosing or alternating schedules rather than continuous MTD to limit selection of double-resistant clones. Although dual resistance is initially rare, sustained selective pressure will enrich these populations, so even effective combinations should be deployed strategically.

### 6.4. Extinction Therapy

Extinction therapy is an evolutionarily informed strategy that aims to eradicate cancer by driving tumor cell populations below a critical threshold from which recovery is impossible, drawing on principles from ecological extinction dynamics [56,57]. Unlike traditional MTD approaches or adaptive therapy, extinction therapy uses sequential therapeutic perturbations that exploit the vulnerabilities of small, declining cancer populations [56,57,58].

The extinction therapy approach is based on observations from Anthropocene extinctions (human-caused species extinctions) rather than catastrophic mass extinctions. The strategy involves a “first strike–second strike” sequence where the first strike, or initial therapy, reduces tumor population size and genetic diversity; then the second strike exploits the eco-evolutionary vulnerability of the now-small, fragmented populations, driving them below the minimum viable population threshold [57]. This creates an “extinction vortex” where small populations become increasingly vulnerable to stochastic perturbations, demographic collapse, and loss of adaptive capacity [56].

The key distinction from traditional sequential therapy is that each strike is administered before resistance develops to the prior therapy, preserving the option to return to previously used drug classes if extinction fails and a transition to adaptive therapy becomes necessary [59]. There is a precedent for this in pediatric Acute Lymphoblastic Leukemia in which a highly successful, empirically derived curative therapy has been developed. The initial “induction” treatment represents a first strike followed quickly by a second strike (“treatment intensification”), third strike (“delayed intensification”), and fourth strike (“maintenance”) [57].

This approach maintains flexibility by rotating mechanisms of action while drugs remain effective, avoiding treatment-until-progression that eliminates future options. If cure is not achieved, preserved therapies can be used adaptively to maintain long-term control [59]. This strategic sequencing creates a “safety net” where failure of the extinction attempt does not preclude long-term disease control through evolutionary management.

The distinguishing feature from standard consolidation or empirical drug rotation is that each ‘strike’ is timed proactively to the eco-evolutionary vulnerability of small, declining populations—before resistance to the prior agent emerges—with the explicit intent of driving populations below a minimum viable threshold while preserving drug classes for later adaptive use [60].

The implication: extinction-therapy principles can be extended from oligometastatic to diffuse MBC, but this is an unproven hypothesis at this time; breast-specific clinical evidence for extinction therapy as an eradication-oriented strategy is currently absent. However, prospective trials are underway. At Moffitt Cancer Center, a phase II trial in HR+/HER2– MBC (NCT06409390, registry date 7 May 2024) tests sequential strikes with cyclophosphamide/docetaxel, antibody–drug conjugates, and capecitabine. A prostate cancer phase II trial (NCT05189457, registry date 29 December 2021) evaluates sequential androgen deprivation, novel hormonal agents, and docetaxel. A multi-institutional trial testing this approach is underway in metastatic Ewing’s Sarcoma (NCT07194044).

Additional eco-evolutionary extinction trials are in development in breast cancer (NCT06439693, registry date 26 September 2025), translating extinction principles into prospective clinical testing. Ongoing trials should be regarded as exploratory and hypothesis-driven.

### 6.5. Biomarker Monitoring

Traditional monitoring in MBC relies on imaging or tumor markers every 2–4 months. While these tools detect progression, they identify resistance after resistant clones have expanded to cause measurable growth—often too late to intervene effectively [61,62].

Circulating tumor DNA (ctDNA) offers tracking of tumor evolution in real time, potentially detecting resistance months before radiographic progression. ctDNA consists of tumor-derived DNA fragments circulating in blood, carrying the same mutations present in tumor cells [63,64]. By sequencing ctDNA serially, we can track the emergence and expansion of resistant clones, monitor treatment response at the molecular level, and potentially adjust therapy before clinical progression [62,65].

Growing evidence supports its role in breast cancer. ctDNA clearance predicts improved outcomes, while persistent or rising levels predict progression [66,67]. In the plasmaMATCH trial, low baseline ctDNA and on-treatment clearance correlated with longer PFS and higher response rates [63]. ctDNA can detect resistance mutations—such as ESR1 and PIK3CA—months before imaging [64,68]. In the PADA-1 and SERENA-6 breast cancer trials, switching therapy based on ESR1 mutations in ctDNA improved PFS [62,69].

Beyond specific mutations, ctDNA diversity and variant allele frequency reflect clonal dynamics: increasing diversity suggests emerging resistance, while decreasing levels indicate effective control [67,70].

Thus, ctDNA is not merely an earlier progression marker—it provides a window into tumor evolutionary state, helping guide adaptive treatment decisions [62,71].

ctDNA is a promising monitoring modality with growing evidence, but its use to time treatment pauses and resumption remains largely trial-driven and is not established for routine practice. Interpretation is limited by assay sensitivity and shedding-dependent false negatives, false positives (e.g., clonal hematopoiesis), preanalytical and analytical variability, lack of assay standardization, and cost and access constraints. Serial ctDNA can detect molecular progression a median of several months before imaging, which is the basis for prospective ctDNA-guided strategies now under evaluation. As eco-evolutionary strategies get incorporated in clinical trials, ctDNA can help time treatment pauses and resumption based on tumor dynamics rather than fixed schedules and give a window of opportunity to assess emerging mutations that can be used for treatment selection.

### 6.6. Real-World Examples

Several real-world and trial-embedded observations suggest that explicitly accounting for evolutionary dynamics—rather than pursuing continuous maximal tumor suppression—can meaningfully influence outcomes in MBC. In HR+ HER2 negative disease, adaptive dosing strategies and planned treatment de-escalation have emerged from both prospective and retrospective analyses. Studies such as the SONIA trial could suggest that delaying or intermittently withholding CDK4/6 inhibitors in selected patients does not uniformly compromise outcomes and may possibly reduce selective pressure for resistant clones, challenging the paradigm of continuous combination therapy until progression [72]. The SERENA-6 trial demonstrated a paradigm shift from imaging-driven to molecularly driven treatment adaptation. Rather than waiting for resistant clones to proliferate into radiologically detectable masses, ctDNA monitoring enabled a “first strike–second strike” approach: the initial aromatase inhibitor plus CDK4/6 inhibitor combination provides the first strike that reduces tumor burden, while switching to camizestrant upon ESR1 mutation detection provides the second strike that targets the emerging resistant population before it can establish dominance [59]. The dramatic improvement in 24-month PFS (29.7% vs. 5.4%) and delayed quality-of-life deterioration suggest that this strategy successfully prevents the evolutionary trajectory toward treatment failure [59,69].

In HER2+ MBC, long-term disease control has been observed in a subset of patients maintained on trastuzumab-based therapy, often combined with endocrine treatment, without continuous chemotherapy. Clinical experience and registry data show that some patients remain progression-free for years on maintenance HER2 blockade, an observational clinical pattern that is consistent with, but not proven to be caused by, eco-evolutionary stabilization of the tumor ecosystem. This raises the possibility that sustained suppression of HER2-dependent clones—without aggressive cytotoxic pressure—may stabilize tumor ecosystems rather than drive rapid resistance [19].

### 6.7. Incorporating Eco-Evolutionary Modeling into the Clinical Pipeline

Cancer treatment decisions in metastatic disease are often made in the setting of ongoing biological change. Tumors are heterogeneous, adapt to therapy, and may respond well initially before progressing. Eco-evolutionary modeling provides a structured way to interpret these dynamics and support time-dependent clinical decisions.

The process begins with a clinical question—when to modify therapy, whether a response is durable, or how to balance suppression with resistance risk. Rather than focusing on average outcomes, eco-evolutionary models account for how tumor subpopulations respond over time. They use routine longitudinal data—imaging, ctDNA, serum markers—as indicators of tumor behavior, allowing clinicians to interpret changes over time rather than relying on single time-point assessments, without requiring additional invasive testing.

**Figure 2:**
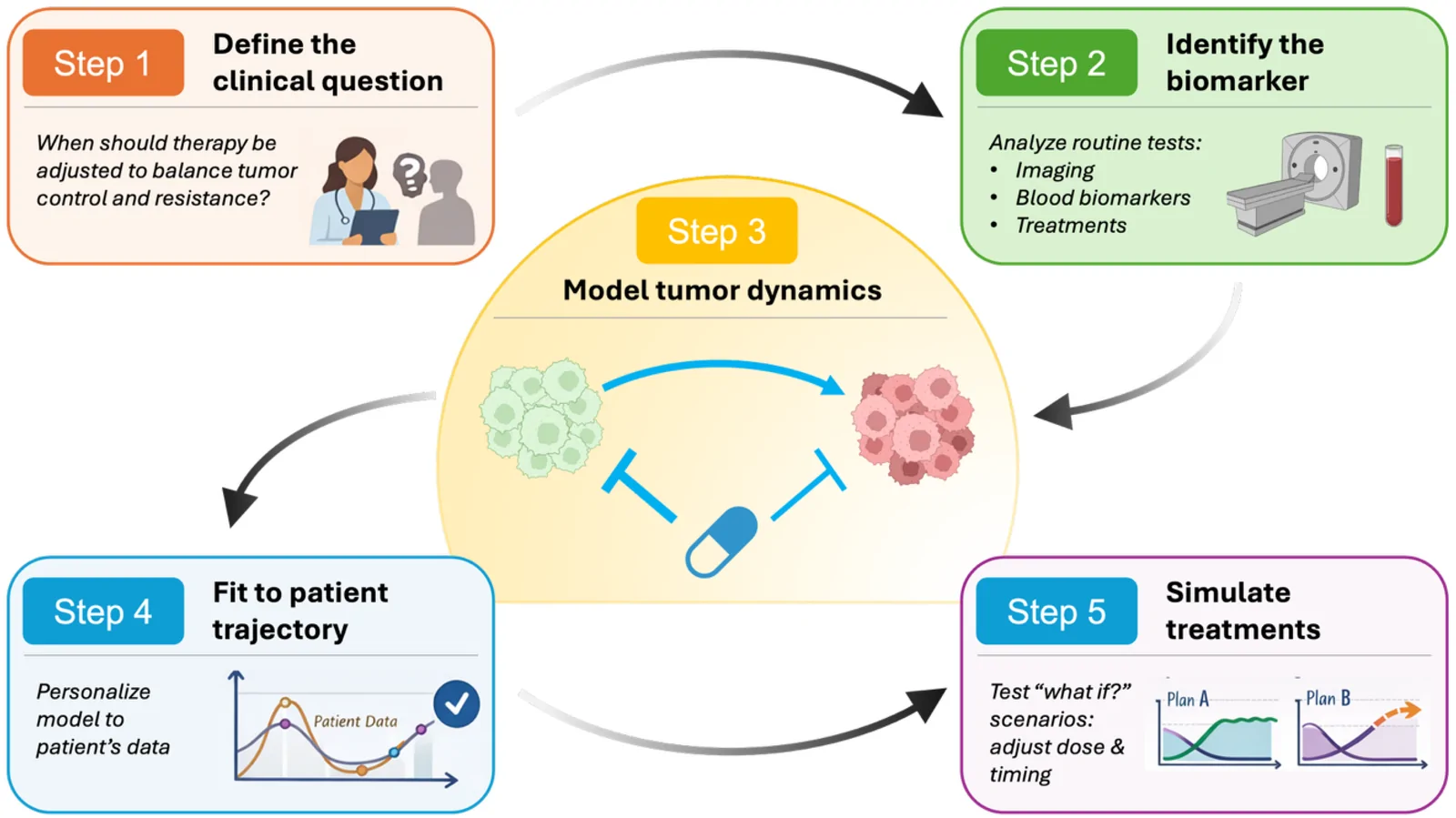
Eco-evolutionary modeling workflow for adaptive cancer therapy. Beginning from clinically relevant questions, longitudinal biomarkers are used to track tumor burden over time. Tumor dynamics are represented by treatment-sensitive and treatment-resistant subpopulations, both suppressed by systemic therapy but with stronger effect on sensitive cells. Solid blue lines indicate therapeutic inhibition, with thickness scaling to the magnitude of suppression—hence the thicker line on the sensitive population. After fitting to patient data, the model is used to simulate alternative treatment strategies.

Tumor dynamics are represented intuitively but in a structured way: therapy suppresses sensitive cells while resistant cells may persist and drive progression (Figure 1). With patient-specific data, models can be calibrated to observed trajectories and used to simulate alternative strategies. By testing “what if” scenarios in silico, clinicians can explore treatment schedules that may delay resistance and prolong control (Figure 2).

Insights from eco-evolutionary-inspired clinical trials in prostate cancer (NCT03511196) [42], advanced melanoma (NCT03543969), and thyroid cancer (NCT03630120 https://clinicaltrials.gov/study/NCT03630120), for accessed on 25 May 2026 instance, have set the stage for a new generation of clinical trials that explicitly incorporate adaptive or extinction-based strategies grounded in eco-evolutionary theory into the breast cancer space. A pilot clinical trial investigating an extinction-informed approach in patients with HR+, HER2 negative MBC is currently open and enrolling, with a parallel focus on identifying biomarkers of response and resistance (NCT06409390, registry date 7 May 2024). In parallel, another study is evaluating a sequential treatment strategy with curative intent in patients with de novo HER2+ MBC, challenging the traditional assumption that continuous maximal therapy is required for durable disease control (NCT06439693). Finally, an adaptive therapy trial using capecitabine in metastatic ER+ HER2 negative breast cancer directly tests dose modulation based on disease dynamics rather than fixed schedules (NCT06525766). Collectively, these trials represent an important shift from static treatment paradigms toward dynamic, evolution-informed strategies aimed at prolonging disease control and delaying therapeutic resistance to potentially change how we treat patients in the future.

## 7. Limitations

Resistance arising from rapid phenotypic plasticity rather than stable genetic mutations can enable cancer cells to reversibly switch between sensitive and resistant states, thereby escaping the competitive suppression that adaptive therapy relies upon; drug-sensitive and drug-resistant phenotypes can interchange, reducing the benefits of adaptive therapy compared to continuous treatment [73,74].

In highly aggressive disease, the rapid tumor growth kinetics may not provide sufficient time for the monitoring and dose adjustments that adaptive therapy requires, particularly when aggressive resistant subpopulations expand quickly. This could be the case in aggressive TNBC subtypes or in breast cancer that recurs quickly after curative treatment. In such cases, adaptive therapy may fail to outperform MTD approaches, necessitating a switch to high-frequency administration or alternative strategies [75].

Additionally, adaptive therapy’s efficacy depends critically on a fitness cost of resistance that allows drug-sensitive cells to outcompete resistant cells in the absence of treatment, but this competitive advantage can be limited or eliminated by microenvironmental factors; the tumor microenvironment—including cancer-associated fibroblasts, hypoxia, stromal support, and immunosuppressive elements—can provide metabolic and survival advantages to resistant cells, effectively subsidizing their fitness costs and enabling them to proliferate even when treatment-sensitive cells are present, thereby undermining the competitive suppression mechanism central to adaptive therapy [76,77,78].

## 8. Conclusions

Despite major therapeutic advances, MBC continues to follow a predictable pattern: initial response followed by acquired resistance and progression. This reflects not therapeutic failure, but the evolutionary nature of cancer as a heterogeneous ecosystem shaped by selective pressure. Continuous MTD therapy may inadvertently accelerate resistance by eliminating sensitive cells and enabling competitive release of resistant clones.

Eco-evolutionary principles provide a complementary framework that reframes resistance as an expected outcome of Darwinian selection. Strategies such as adaptive dosing, treatment modulation, and biologically informed sequencing aim to preserve intratumoral competition and exploit fitness costs of resistance, shifting the goal from eradication to durable control. Emerging clinical data and advances in longitudinal monitoring, particularly circulating tumor DNA, make evolution-informed treatment increasingly feasible.

Although these approaches should currently remain within clinical trials, ongoing prospective studies will define their role in routine care. The promise of eco-evolutionary oncology is not less therapy, but smarter therapy—aligning treatment with tumor biology to achieve more sustained disease control over a longer time.

The evolutionary perspective outlined here should therefore be understood as a complementary lens that generates testable hypotheses—identifying which patients, tumor subtypes, and resistance mechanisms might benefit from strategies that preserve competitive suppression—rather than as a basis for changing current practice. These hypotheses warrant prospective evaluation in appropriately designed clinical trials to determine whether they can meaningfully improve outcomes.

## Data Availability

No new data were created or analyzed in this study; data sharing is not applicable. Figure 1 and Figure 2 are schematic, model-based illustrations and do not derive from primary patient datasets.

## Abbreviations

The following abbreviations are used in this manuscript:

ctDNA	Circulating tumor deoxyribonucleic acid
CDK4/6	Cyclin-dependent kinases 4 and 6
DNA	Deoxyribonucleic acid
ESR1	Estrogen receptor 1
HER2	Human epidermal growth factor receptor 2
HR	Hormone receptor
MBC	Metastatic breast cancer
MTD	Maximum tolerated dose
OS	Overall survival
PFS	Progression-free survival
PIK3	Phosphoinositide 3 kinase
RB1	Retinoblastoma 1
TNBC	Triple-negative breast cancer

## References

[1] Goetz M.P., Toi M., Campone M., Sohn J., Paluch-Shimon S., Huober J., Park I.H., Tredan O., Chen S.C., Manso L. (2017). MONARCH 3: Abemaciclib as Initial Therapy for Advanced Breast Cancer. J. Clin. Oncol..

[2] Hortobagyi G.N., Stemmer S.M., Burris H.A., Yap Y.S., Sonke G.S., Paluch-Shimon S., Campone M., Petrakova K., Blackwell K.L., Winer E.P. (2018). Updated results from MONALEESA-2, a phase III trial of first-line ribociclib plus letrozole versus placebo plus letrozole in hormone receptor-positive, HER2-negative advanced breast cancer. Ann. Oncol..

[3] Finn R.S., Martin M., Rugo H.S., Jones S., Im S.A., Gelmon K., Harbeck N., Lipatov O.N., Walshe J.M., Moulder S. (2016). Palbociclib and Letrozole in Advanced Breast Cancer. N. Engl. J. Med..

[4] Swain S.M., Miles D., Kim S.B., Im Y.H., Im S.A., Semiglazov V., Ciruelos E., Schneeweiss A., Loi S., Monturus E. (2020). Pertuzumab, trastuzumab, and docetaxel for HER2-positive metastatic breast cancer (CLEOPATRA): End-of-study results from a double-blind, randomised, placebo-controlled, phase 3 study. Lancet Oncol..

[5] Caswell-Jin J.L., Sun L.P., Munoz D., Lu Y., Li Y., Huang H., Hampton J.M., Song J., Jayasekera J., Schechter C. (2024). Analysis of Breast Cancer Mortality in the US-1975 to 2019. JAMA.

[6] Taskindoust M., Thomas S.M., Sammons S.L., Fayanju O.M., DiLalla G., Hwang E.S., Plichta J.K. (2021). Survival Outcomes Among Patients with Metastatic Breast Cancer: Review of 47,000 Patients. Ann. Surg. Oncol..

[7] Burstein H.J. (2020). Systemic Therapy for Estrogen Receptor-Positive, HER2-Negative Breast Cancer. N. Engl. J. Med..

[8] Spring L.M., Wander S.A., Andre F., Moy B., Turner N.C., Bardia A. (2020). Cyclin-dependent kinase 4 and 6 inhibitors for hormone receptor-positive breast cancer: Past, present, and future. Lancet.

[9] Kannan K., Srinivasan A., Kannan A., Ali N. (2025). The Underlying Mechanisms and Emerging Strategies to Overcome Resistance in Breast Cancer. Cancers.

[10] Roszkowska M. (2024). Multilevel Mechanisms of Cancer Drug Resistance. Int. J. Mol. Sci..

[11] Enriquez-Navas P.M., Wojtkowiak J.W., Gatenby R.A. (2015). Application of Evolutionary Principles to Cancer Therapy. Cancer Res..

[12] Gatenby R.A., Silva A.S., Gillies R.J., Frieden B.R. (2009). Adaptive therapy. Cancer Res..

[13] Newton P.K., Ma Y. (2019). Nonlinear adaptive control of competitive release and chemotherapeutic resistance. Phys. Rev. E.

[14] West J., Ma Y., Newton P.K. (2018). Capitalizing on competition: An evolutionary model of competitive release in metastatic castration resistant prostate cancer treatment. J. Theor. Biol..

[15] Gedye C., Navani V. (2022). Find the path of least resistance: Adaptive therapy to delay treatment failure and improve outcomes. Biochim. Biophys. Acta Rev. Cancer.

[16] West J., Adler F., Gallaher J., Strobl M., Brady-Nicholls R., Brown J., Roberson-Tessi M., Kim E., Noble R., Viossat Y. (2023). A survey of open questions in adaptive therapy: Bridging mathematics and clinical translation. eLife.

[17] Kuss J.T., Muss H.B., Hoen H., Case L.D. (1997). Tamoxifen as initial endocrine therapy for metastatic breast cancer: Long term follow-up of two Piedmont Oncology Association (POA) trials. Breast Cancer Res. Treat..

[18] Andre F., Hee Park Y., Kim S.B., Takano T., Im S.A., Borges G., Lima J.P., Aksoy S., Gavila Gregori J., De Laurentiis M. (2023). Trastuzumab deruxtecan versus treatment of physician’s choice in patients with HER2-positive metastatic breast cancer (DESTINY-Breast02): A randomised, open-label, multicentre, phase 3 trial. Lancet.

[19] Baselga J., Cortes J., Kim S.B., Im S.A., Hegg R., Im Y.H., Roman L., Pedrini J.L., Pienkowski T., Knott A. (2012). Pertuzumab plus trastuzumab plus docetaxel for metastatic breast cancer. N. Engl. J. Med..

[20] Verma S., Miles D., Gianni L., Krop I.E., Welslau M., Baselga J., Pegram M., Oh D.Y., Dieras V., Guardino E. (2012). Trastuzumab emtansine for HER2-positive advanced breast cancer. N. Engl. J. Med..

[21] Bardia A., Hurvitz S.A., Tolaney S.M., Loirat D., Punie K., Oliveira M., Brufsky A., Sardesai S.D., Kalinsky K., Zelnak A.B. (2021). Sacituzumab Govitecan in Metastatic Triple-Negative Breast Cancer. N. Engl. J. Med..

[22] Schmid P., Cortes J., Pusztai L., McArthur H., Kummel S., Bergh J., Denkert C., Park Y.H., Hui R., Harbeck N. (2020). Pembrolizumab for Early Triple-Negative Breast Cancer. N. Engl. J. Med..

[23] Greaves M., Maley C.C. (2012). Clonal evolution in cancer. Nature.

[24] Dagogo-Jack I., Shaw A.T. (2018). Tumour heterogeneity and resistance to cancer therapies. Nat. Rev. Clin. Oncol..

[25] Marusyk A., Janiszewska M., Polyak K. (2020). Intratumor Heterogeneity: The Rosetta Stone of Therapy Resistance. Cancer Cell.

[26] Aleksakhina S.N., Kashyap A., Imyanitov E.N. (2019). Mechanisms of acquired tumor drug resistance. Biochim. Biophys. Acta Rev. Cancer.

[27] Goyette M.A., Lipsyc-Sharf M., Polyak K. (2023). Clinical and translational relevance of intratumor heterogeneity. Trends Cancer.

[28] Park J., Newton P.K. (2023). Stochastic competitive release and adaptive chemotherapy. Phys. Rev. E.

[29] Gallaher J.A., Enriquez-Navas P.M., Luddy K.A., Gatenby R.A., Anderson A.R.A. (2018). Spatial Heterogeneity and Evolutionary Dynamics Modulate Time to Recurrence in Continuous and Adaptive Cancer Therapies. Cancer Res..

[30] Kareva I. (2022). Different costs of therapeutic resistance in cancer: Short- and long-term impact of population heterogeneity. Math. Biosci..

[31] Silva A.S., Kam Y., Khin Z.P., Minton S.E., Gillies R.J., Gatenby R.A. (2012). Evolutionary approaches to prolong progression-free survival in breast cancer. Cancer Res..

[32] Bacevic K., Noble R., Soffar A., Wael Ammar O., Boszonyik B., Prieto S., Vincent C., Hochberg M.E., Krasinska L., Fisher D. (2017). Spatial competition constrains resistance to targeted cancer therapy. Nat. Commun..

[33] Hockings H., Lakatos E., Huang W., Mossner M., Khan M.A., Bakali N., McDermott J., Smith K., Baker A.M., Graham T.A. (2025). Adaptive Therapy Exploits Fitness Deficits in Chemotherapy-Resistant Ovarian Cancer to Achieve Long-Term Tumor Control. Cancer Res..

[34] Wodarz D. (2021). Adaptive Therapy and the Cost of Drug-Resistant Mutants. Cancer Res..

[35] Kim E., Brown J.S., Eroglu Z., Anderson A.R.A. (2021). Adaptive Therapy for Metastatic Melanoma: Predictions from Patient Calibrated Mathematical Models. Cancers.

[36] West J., You L., Zhang J., Gatenby R.A., Brown J.S., Newton P.K., Anderson A.R.A. (2020). Towards Multidrug Adaptive Therapy. Cancer Res..

[37] Thomas D.S., Cisneros L.H., Anderson A.R.A., Maley C.C. (2022). In Silico Investigations of Multi-Drug Adaptive Therapy Protocols. Cancers.

[38] Zhang L., Ma J., Liu L., Li G., Li H., Hao Y., Zhang X., Ma X., Chen Y., Wu J. (2023). Adaptive therapy: A tumor therapy strategy based on Darwinian evolution theory. Crit. Rev. Oncol. Hematol..

[39] Gluzman M., Scott J.G., Vladimirsky A. (2020). Optimizing adaptive cancer therapy: Dynamic programming and evolutionary game theory. Proc. R. Soc. B Biol. Sci..

[40] Seyedi S., Teo R., Foster L., Saha D., Mina L., Northfelt D., Anderson K.S., Shibata D., Gatenby R., Cisneros L.H. (2024). Testing Adaptive Therapy Protocols Using Gemcitabine and Capecitabine in a Preclinical Model of Endocrine-Resistant Breast Cancer. Cancers.

[41] Brady-Nicholls R., Enderling H. (2022). Range-Bounded Adaptive Therapy in Metastatic Prostate Cancer. Cancers.

[42] Zhang J., Gallaher J., Cunningham J.J., Choi J.W., Ionescu F., Chatwal M.S., Jain R., Kim Y., Wang L., Brown J.S. (2022). A Phase 1b Adaptive Androgen Deprivation Therapy Trial in Metastatic Castration Sensitive Prostate Cancer. Cancers.

[43] Crucitta S., Cucchiara F., Mathijssen R., Mateo J., Jager A., Joosse A., Passaro A., Attili I., Petrini I., van Schaik R. (2022). Treatment-driven tumour heterogeneity and drug resistance: Lessons from solid tumours. Cancer Treat. Rev..

[44] Acar A., Nichol D., Fernandez-Mateos J., Cresswell G.D., Barozzi I., Hong S.P., Trahearn N., Spiteri I., Stubbs M., Burke R. (2020). Exploiting evolutionary steering to induce collateral drug sensitivity in cancer. Nat. Commun..

[45] Zhao B., Sedlak J.C., Srinivas R., Creixell P., Pritchard J.R., Tidor B., Lauffenburger D.A., Hemann M.T. (2016). Exploiting Temporal Collateral Sensitivity in Tumor Clonal Evolution. Cell.

[46] Hall M.D., Handley M.D., Gottesman M.M. (2009). Is resistance useless? Multidrug resistance and collateral sensitivity. Trends Pharmacol. Sci..

[47] Pluchino K.M., Hall M.D., Goldsborough A.S., Callaghan R., Gottesman M.M. (2012). Collateral sensitivity as a strategy against cancer multidrug resistance. Drug Resist. Updat..

[48] Efferth T., Saeed M.E.M., Kadioglu O., Seo E.J., Shirooie S., Mbaveng A.T., Nabavi S.M., Kuete V. (2020). Collateral sensitivity of natural products in drug-resistant cancer cells. Biotechnol. Adv..

[49] Qian J., Cui J., Li S., Chen J., Jia J. (2021). Anticancer Natural Products with Collateral Sensitivity: A Review. Mini Rev. Med. Chem..

[50] Dhawan A., Nichol D., Kinose F., Abazeed M.E., Marusyk A., Haura E.B., Scott J.G. (2017). Collateral sensitivity networks reveal evolutionary instability and novel treatment strategies in ALK mutated non-small cell lung cancer. Sci. Rep..

[51] Gottesman M.M., Lavi O., Hall M.D., Gillet J.P. (2016). Toward a Better Understanding of the Complexity of Cancer Drug Resistance. Annu. Rev. Pharmacol. Toxicol..

[52] Scheuer W., Friess T., Burtscher H., Bossenmaier B., Endl J., Hasmann M. (2009). Strongly enhanced antitumor activity of trastuzumab and pertuzumab combination treatment on HER2-positive human xenograft tumor models. Cancer Res..

[53] Rosenheim J.A. (2018). Short- and long-term evolution in our arms race with cancer: Why the war on cancer is winnable. Evol. Appl..

[54] Marine J.C., Dawson S.J., Dawson M.A. (2020). Non-genetic mechanisms of therapeutic resistance in cancer. Nat. Rev. Cancer.

[55] Niu X., Liu W., Zhang Y., Liu J., Zhang J., Li B., Qiu Y., Zhao P., Wang Z., Wang Z. (2024). Cancer plasticity in therapy resistance: Mechanisms and novel strategies. Drug Resist. Updat..

[56] Gatenby R.A., Artzy-Randrup Y., Epstein T., Reed D.R., Brown J.S. (2020). Eradicating Metastatic Cancer and the Eco-Evolutionary Dynamics of Anthropocene Extinctions. Cancer Res..

[57] Gatenby R.A., Zhang J., Brown J.S. (2019). First Strike-Second Strike Strategies in Metastatic Cancer: Lessons from the Evolutionary Dynamics of Extinction. Cancer Res..

[58] Felder S.I., Fleming J.B., Gatenby R.A. (2021). Treatment-induced evolutionary dynamics in nonmetastatic locally advanced rectal adenocarcinoma. Adv. Cancer Res..

[59] Bidard F.C., Mayer E.L., Park Y.H., Janni W., Ma C., Cristofanilli M., Bianchini G., Kalinsky K., Iwata H., Chia S. (2025). First-Line Camizestrant for Emerging ESR1-Mutated Advanced Breast Cancer. N. Engl. J. Med..

[60] Strobl M.A.R., West J., Viossat Y., Damaghi M., Robertson-Tessi M., Brown J.S., Gatenby R.A., Maini P.K., Anderson A.R.A. (2021). Turnover Modulates the Need for a Cost of Resistance in Adaptive Therapy. Cancer Res..

[61] Merker J.D., Oxnard G.R., Compton C., Diehn M., Hurley P., Lazar A.J., Lindeman N., Lockwood C.M., Rai A.J., Schilsky R.L. (2018). Circulating Tumor DNA Analysis in Patients with Cancer: American Society of Clinical Oncology and College of American Pathologists Joint Review. J. Clin. Oncol..

[62] Valenza C., Trapani D., Curigliano G. (2022). Circulating tumour DNA dynamics for assessment of molecular residual disease and for intercepting resistance in breast cancer. Curr. Opin. Oncol..

[63] Browne I.M., Pascual J., Cutts R.J., Kingston B., Hrebien S., Kilburn L.S., Pearson A., Moretti L., Wardley A.M., Macpherson I.R. (2026). The Prognostic and Predictive Impact of ctDNA Levels in Patients with Advanced Breast Cancer Enrolled on the plasmaMATCH Trial. Clin. Cancer Res..

[64] Tegeler C.M., Hartkopf A.D., Banys-Paluchowski M., Krawczyk N., Fehm T., Jaeger B.A.S. (2024). Circulating Tumor DNA in Early and Metastatic Breast Cance-Current Role and What Is Coming Next. Cancers.

[65] Xu J., Gao H., Guan X., Meng J., Ding S., Long Q., Yi W. (2024). Circulating tumor DNA: From discovery to clinical application in breast cancer. Front. Immunol..

[66] Elliott M.J., Echelard P., Pipinikas C., Main S., Fuentes Antras J., Dou A., Veitch Z., Amir E., Nadler M.B., Meti N. (2025). Longitudinal evaluation of circulating tumor DNA in patients undergoing neoadjuvant therapy for early breast cancer using a tumor-informed assay. Nat. Commun..

[67] Magbanua M.J.M., Manon N.A., Wolf D.M., Rivero-Hinojosa S., Ahmed Z., Sayaman R.W., Tin A., Renner D., Kalashnikova E., Brown-Swigart L. (2025). Circulating tumor DNA refines risk stratification of neoadjuvant therapy-resistant breast tumors. Nat. Commun..

[68] Jacob S., Davis A.A., Gerratana L., Velimirovic M., Shah A.N., Wehbe F., Katam N., Zhang Q., Flaum L., Siziopikou K.P. (2021). The Use of Serial Circulating Tumor DNA to Detect Resistance Alterations in Progressive Metastatic Breast Cancer. Clin. Cancer Res..

[69] Turner N.C., Mayer E.L., Park Y.H., Janni W., Ma C., Cristofanilli M., Bianchini G., Kalinsky K., Iwata H., Chia S. (2026). Switching to camizestrant at ESR1 mutation emergence before disease progression during first-line treatment of hormone receptor-positive advanced breast cancer (SERENA-6): Extended analysis of a double-blind, placebo-controlled, randomised, phase 3 trial. Lancet Oncol..

[70] Liu J., Fang F., Yuan W., Chen T., Wang J., Miao X., Meng Y., Song C., Tan W., An J. (2026). Integrating ctDNA Dynamics and Sequential Organoid Drug Screening Enhances Breast Cancer Treatment Efficacy. Cancer Res..

[71] Loizidis S., Stanciu M.A., Ignatiadis M. (2025). Liquid biopsy in breast cancer: Promises and challenges. Curr. Opin. Oncol..

[72] Sonke G.S., van Ommen-Nijhof A., Wortelboer N., van der Noort V., Swinkels A.C.P., Blommestein H.M., Guerrero Paez C., Mol L., Beeker A., Beelen K. (2024). Early versus deferred use of CDK4/6 inhibitors in advanced breast cancer. Nature.

[73] Masud M.A., Kim J.Y., Kim E. (2023). Modeling the effect of acquired resistance on cancer therapy outcomes. Comput. Biol. Med..

[74] Vibishan B., Jain P., Sharma V., Hari K., Kadelka C., George J.T., Jolly M.K. (2025). Impacts of competition and phenotypic plasticity on the viability of adaptive therapy. Math. Biosci..

[75] Wang J., Zhang Y., Liu X., Liu H. (2021). Optimizing Adaptive Therapy Based on the Reachability to Tumor Resistant Subpopulation. Cancers.

[76] Al-Akra L., Bae D.H., Leck L.Y.W., Richardson D.R., Jansson P.J. (2019). The biochemical and molecular mechanisms involved in the role of tumor micro-environment stress in development of drug resistance. Biochim. Biophys. Acta Gen. Subj..

[77] Erin N., Grahovac J., Brozovic A., Efferth T. (2020). Tumor microenvironment and epithelial mesenchymal transition as targets to overcome tumor multidrug resistance. Drug Resist. Updat..

[78] Valcz G., Gatenby R.A., Ujvari B., Buzas E.I., Molnar B. (2025). Adaptive cancer therapy: Can non-genetic factors become its achilles heel?. Oncogene.

